# The cytotoxicity and apoptotic effects of verbascoside on breast cancer 4T1 cell line

**DOI:** 10.1186/s40360-021-00540-8

**Published:** 2021-11-29

**Authors:** Atena Daneshforouz, Samad Nazemi, Omid Gholami, Marzieh Kafami, Bahareh Amin

**Affiliations:** 1 Student Research Center, Sabzevar University of Medical Sciences, Sabzevar, Iran; 2 Cellular and Molecular Research Center, Department of Physiology and Pharmacology, Faculty of Medicine, Sabzevar University of Medical Sciences, Sabzevar, Iran; 3 Cellular and Molecular Research Center, Sabzevar University of Medical Sciences, Sabzevar, Iran; 4 Department of Physiology and Pharmacology, Faculty of Medicine, Sabzevar University of Medical Sciences, Sabzevar, Iran

**Keywords:** Verbascoside, Breast cancer, Apoptosis, Toll like receptor, *MyD88*, *NF-κB*, Caspase-3

## Abstract

**Background:**

Despite significant advancements in breast cancer therapy, novel drugs with lower side effects are still being demanded. In this regard, we investigated the anti-cancer features of verbascoside in 4 T1 mouse mammary tumor cell.

**Methods:**

First, MTT assay was performed with various concentrations (ranging between 5 to 200 μM) of verbascoside and IC50 was calculated. Then the expression of *Bax*, *Bcl-2*, and *caspase-3* was evaluated in treated 4 T1 cells. In addition, we investigated the expression of *TLR4*, *MyD88*, and *NF-κB* to ascertain the underlying mechanism of the anti-proliferative feature of verbascoside. Also, flow cytometry followed by double PI and Annexin V was conducted to confirm the apoptosis-inducing effect of verbascoside.

**Results:**

Our results from MTT assay showed verbascoside inhibits proliferation of 4 T1 cancer cells (IC50 117 μM) while is safe for normal HEK293T cells. By qRT-PCR, we observed that verbascoside treatment (100, 117 and, 130 μM) increases the expression of *caspase-3* and *Bax* while reduces the expression of *Bcl-2*. Also, verbascoside (100, 117 and, 130 μM) increased the expression of *TLR4* only at 130 μM dose and the expression of *MyD88* whereas reduced the expression of *NF-κB* at mRNA level. Flow cytometry analysis also confirmed verbascoside induces apoptosis in 4 T1 cells at 117 μM.

**Conclusion:**

Taken together, our data showed verbascoside is a safe natural compound for normal cells while has apoptosis-inducing feature through TLR4 axis on 4 T1 cells.

## Introduction

Breast cancer is the major form of malignancy and the most fatal type of cancer in women around the world, with an increasing rate of prevalence and mortality [1]. Attainments in breast cancer treatment have provided various therapeutic options including radiotherapy, surgery, and chemotherapy. Chemotherapeutics are mainly nonselective and cause toxicity for healthy organs and normal tissues which consequently lead to cardiac diseases, reproductive system disorders, neuropathy and infection [2]. Signaling pathways play a vital role during the development and progression of diseases, especially in cancer [3], therefore understanding involved mechanisms and therefore identifying novel drugs for treatment is an important goal in cancer research. In this regard, herbal plants with therapeutic effects have become an interesting field of study for cancer researchers [4]. Herbal medicines have shown promising insights as adjuvant therapy and even main treatment drug [5]. To date, the anti-proliferative effect of polyphenols, flavonoids, terpenoids has been reported in breast cancer treatment [6].

Verbascoside is an active ingredient extracted from *Cistanches Herba* of Orobanchaceae family [7]. To date, antinociceptive, antioxidant, anti-inflammatory, as well as protection against Parkinson and Alzheimer’s disease for this component have been reported [7, 8]. This phenolic substance by activating homeodomain-interacting protein kinase (*HIPK*)-*p53* axis is able to reduce the proliferation of colorectal cancer cells and initiates apoptosis [9]. *HIPK* pathway modulates apoptosis by phosphorylating Ser46 activates *p53*, which in turn leads to apoptosis phenomena by regulating Bcl-2-associated X protein (*Bax*). In addition, *p53* by suppressing nuclear factor kappa-light-chain-enhancer of activated B cells (*NF-κB*) pathway induces apoptosis. The *p53* activated by *HIPK* can directly activate caspase to initiate apoptosis [10].

Toll-like receptor 4 (*TLR4*) is an important signaling pathway in inflammation which also plays a role in cancer prevention [11]. Downregulation of *TLR4* is associated with increased tumor formation and metastasis in animal models [12]. *TLR4* activation can boost immune system defense against tumor cells through *MyD88* which leads to activation or maturation of immune cells such as dendritic cells (DCs), macrophages and T cells [11]. Based on this evidence, to date, various therapeutic drugs for activating *TLR4* in cancer treatment has been implemented [13, 14].

Cancer research has shown that caspase-3 mechanism has a strong link with cell death in tumors which has made it a preferred target for cancer treatment [15]. Change in the harmony of B-cell lymphoma 2 (*Bcl-2*) and Bcl-2-associated X protein (*Bax*) ratio is responsible for the activation of Caspase-3 in response to death signals [16]. *Bcl-2* inhibits the activity of *Bax* which is located at the membrane of mitochondria. *Bax* initiates apoptosis by increasing permeability of mitochondria membrane which in turn disrupts its membrane and release of cytochrome C [17]. Increased *Bax/Bcl-2* ratio reflects the condition of increased pro-apoptotic *Bax* to an anti-apoptotic molecule of *Bcl-2* in cancer cells [18].

In the present study, we aimed to investigate the effect of verbascoside on breast cancer cell line *4 T1*.

## Material and methods

The mouse breast cancer cell lines designated as *4 T1* and human embryonic kidney cell lines designated as *HEK293T* were obtained from the Pasture Institute in Tehran,Iran.

### Cell culture and drug preparation

*4 T1* and *HEK293T* cells were cultured in Dulbecco’s Modified Eagle Medium (*DMEM*) and Roswell Park Memorial Institute 1640 (*RPMI 1640*) respectively, supplemented with 10% *FBS* (KalaZist CO) and 1% Penicillin (100 units/ml)/Streptomycin (100 μg/ml). Flasks were incubated at 37 °C in CO_2_ incubator with a 95% humidity. Media was changed every 2 days and at 85% confluency cells were passaged. After the third passage, cells were seeded in proper plates and number for further experiments [19].

The stock solution was made by dissolving 2 mg of verbascoside powder in 100 μl *DMSO* (stock A). Then 50 μl of stock A was added to 950 μl of media to make stock B (corresponding to the type of cells). Treatment concentrations were made from stock B, for instance, 5 μl stock B was added to 995 μl media to make 5 μM concentration and likewise for the higher concentrations. Since the solvent we used was *DMSO*, we also considered a *DMSO* 10% control in 3-[4,5-dimethylthiazole-2-yl]-2,5-diphenyltetrazolium bromide (*MTT*) experiments.

### MTT assay

In order to assess the response of cells to verbascoside at different concentrations and its toxicity, we performed *MTT* assay for both cell lines. 1 × 10^5^ cells per well were seeded into 96 wells flat-bottom plates and incubated to grow for 24 h. Then media was replaced with drug-containing media with a defined concentration of verbascoside ranging between 5 μM to 200 μM, and then cells were incubated for 24 h. After removal of media, *MTT* solution (5 mg/ml in *PBS*) was added to each well and plates were incubated for 4 h. After that, *DMSO* was used to dissolve formazan crystals in wells and absorbance was read at 570 nm (620 nm as the reference wavelength), by an enzyme-linked immunosorbent assay (ELISA) reader instrument. Then, half-maximal inhibitory concentration (IC50(was calculated via GraphPad Prism® [20], and was used for downstream experiments. The experiment was performed in triplicate.

### Gene expression analysis

To perform reverse transcription polymerase chain reaction (RT-PCR), *4 T1* cells were seeded at 3.5 × 10^5^ density in 6-well plates and allowed to grow overnight in the incubator. Next, cells were treated with different concentrations (100, 117 and 130 μm) and incubated for another 24 h. Afterward, RNA extraction from cells was performed via RNX-Plus (Sinaclon, Iran) according to manufacturer protocol. Quality and quantity of RNA samples were checked by Agarose Gel and Nanodrop BIO INTELLECTICA Nano100 (Canada). cDNA synthesis was carried out via RR037Q -Takara (Japan) according to the protocol provided by the manufacturer and with an equal starting nanogram of RNA fro each sample. Real-time PCR was performed by CFX96 Touch™ Bio-Rad (USA) and using SYBR Green® Yekta Tajhiz Azma. The sequence of primers used in this research is reported in Table 1. Relative expression fold changes were calculated through 2^-∆∆CT^ method [21].

**Table 1:** Summary of PCR primer pairs

Name	Sequence of primers	Annealing Temperature
*Bax*	Forward: 5’CAAGGCCCTGTGCACTAAAGT3’	60 °C
	Reverse: 5’AAGTAGGAGAGGAGGCCTTCC3’	
*Bcl2*	Forward: 5’GGAGAAATCAAACAGAGGTCGC3’	60 °C
	Reverse: 5’CGTCAACAGGGAGATGTCACC3’	
*MyD88*	Forward: 5’CTCCAGGTGTCCAACAGAAGC3’	60 °C
	Reverse: 5’TCATCTTCCCCTCTGCCCTAG3’	
*NF-κB*	Forward: 5’GCCATTGAAGTGATCCAGGCA3’	60 °C
	Reverse: 5’TCCCGGAGTTCATCTATGTGCT3’	
*TLR4*	Forward 5’GCATGGATCAGAAAACTCAGC −5’	60 °C
	Reverse: 5’TGTTTCAATTTCACACCTGGA −5’	
*Caspase 3*	Forward: 5’GGAGCAGCTTTGTGTGTGTG3’	60 °C
	Reverse: 5’TCCAGGAATAGTAACCAGGTGC3’	
*GAPDH*	Forward: 5’GGAAGGTGAAGGTCGGAGTCA3’	60 °C
	Reverse: 5’GTCATTGATGGCAACAATATCCACT3’	

### Flow cytometry analysis

Apoptotic cells were detected using propidium iodide (*PI*) and Annexin staining followed by flow cytometry. For this purpose, *4 T1* cells were seeded at 5 × 10^5^ density in wells of 6-well plates and allowed to grow for 24 h. Then media was removed and replaced with media containing 117 μM verbascoside and plates were incubated for another 24 h again. After that time, cells were detached by Trypsin, and staining for Annexin and PI was performed by the protocol described elsewhere [22].

### Statistical analysis

To analyze results from a statistical perspective, we used one-way analysis of variance (ANOVA) and Tukey post-test. All data are expressed as the mean ± SD. *P* values under 0.05 were considered statistically significant.

## Results

### Verbascoside inhibits the growth of 4 T1 cells but not HEK293T cells

**Fig. 1:**
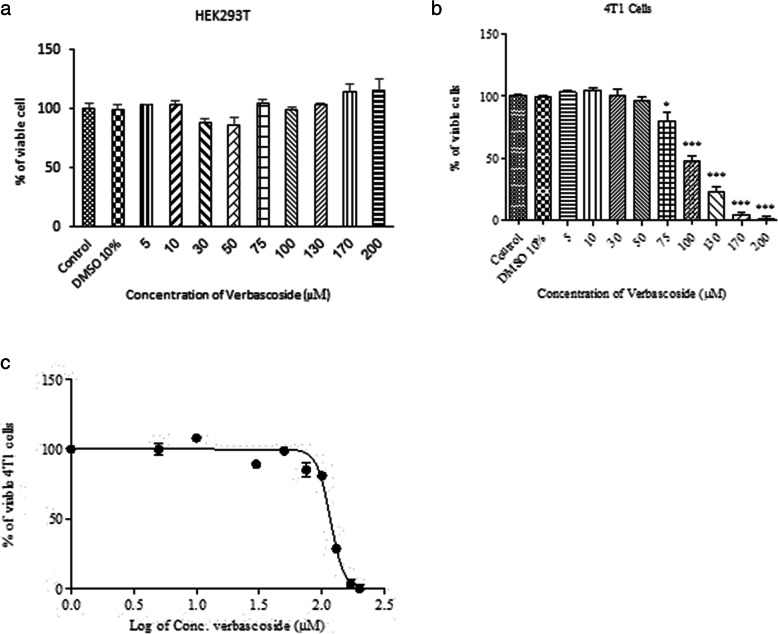
Drug toxicity determination by MTT assay. **a** Verbascoside treatment did not show any toxic effect on the HEK293T cells as a normal control. **b** Verbascoside in a dose-dependent manner inhibited proliferation of 4 T1 cells. The toxic effect was observed from 75 μM concentration (79.75 ± 11.99%) with the highest effect at the 200 μM concentration (0.666 ± 4.9%). **c** the IC50 was calculated 116.5 μM. We used 117 μM concentration in our further experiments as the effective dose. *** *P* < 0.001, ** *P* < 0.01, * *P* < 0.05 compared to control

*4 T1* and *HEK293T* cells were treated with different concentrations of verbascoside (5 to 200 μM), for 24 h. *MTT* assay showed that verbascoside did not affect the viability of *HEK293T* cells, as normal control (Fig. 1-a). On the other hand, verbascoside effectively reduced the viability of *4 T1* cells in a dose-dependent manner (Fig. 1-b). *IC50* was calculated 116.7 (~ 117) μM for verbascoside on *4 T1* cells and was used for further experiments (Fig. 1-c).

### Verbascoside modulates gene expression of the apoptotic pathway

**Fig. 2:**
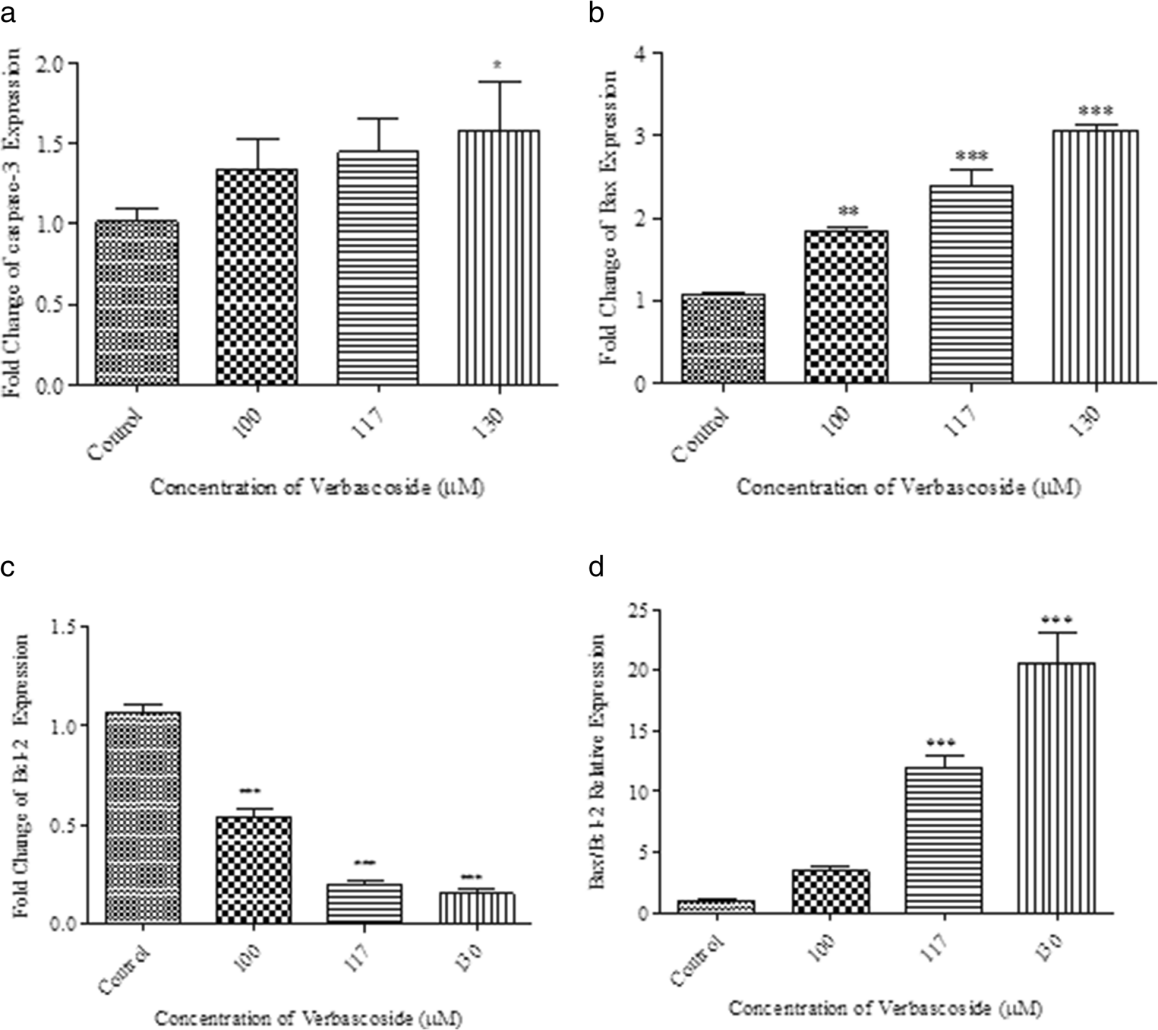
Effect of verbascoside on the expression of genes related to apoptosis in 4 T1 cells. **a** Verbascoside treatment led to the overexpression of caspase3 but was only significant at 130 μM dose. **b** Verbascoside in a dose-dependent manner significantly increased the expression of Bax gene at 100 μM, 117Μm and, 130 μM. **c** Verbascoside significantly reduced the expression of Bcl-2 at 100Μm, 117 μM and,. **d** The effect of verbascoside on the Bax/Bcl-2 ratio. At 100 μM dose ratio (*N* = 3). *** *P* < 0.001, ** *P* < 0.01, * *P* < 0.05 compared to control

qRT-PCR analysis of treated *4 T1* cells demonstrated that verbascoside is able to initiate apoptosis via altering the expression pattern of related genes at the mRNA level. Verbascoside increased the expression of *caspase-3* in a dose-dependent manner (Fig. 2-a). The expression of *Bax* was increased by verbascoside (100 μM *P* < 0.01, 117 μM, 130 μM *P* < 0.001) while the expression of *Bcl-2* was reduced (*P* < 0.001) (Fig. 2-b and -c). In addition, the *Bax/Bcl-2* ratio was increased dose-dependently by verbascoside (Fig. 2-d).

### Verbascosides alters the expression of *MyD88*, *NF-κB* and, *TLR4*

**Fig. 3:**
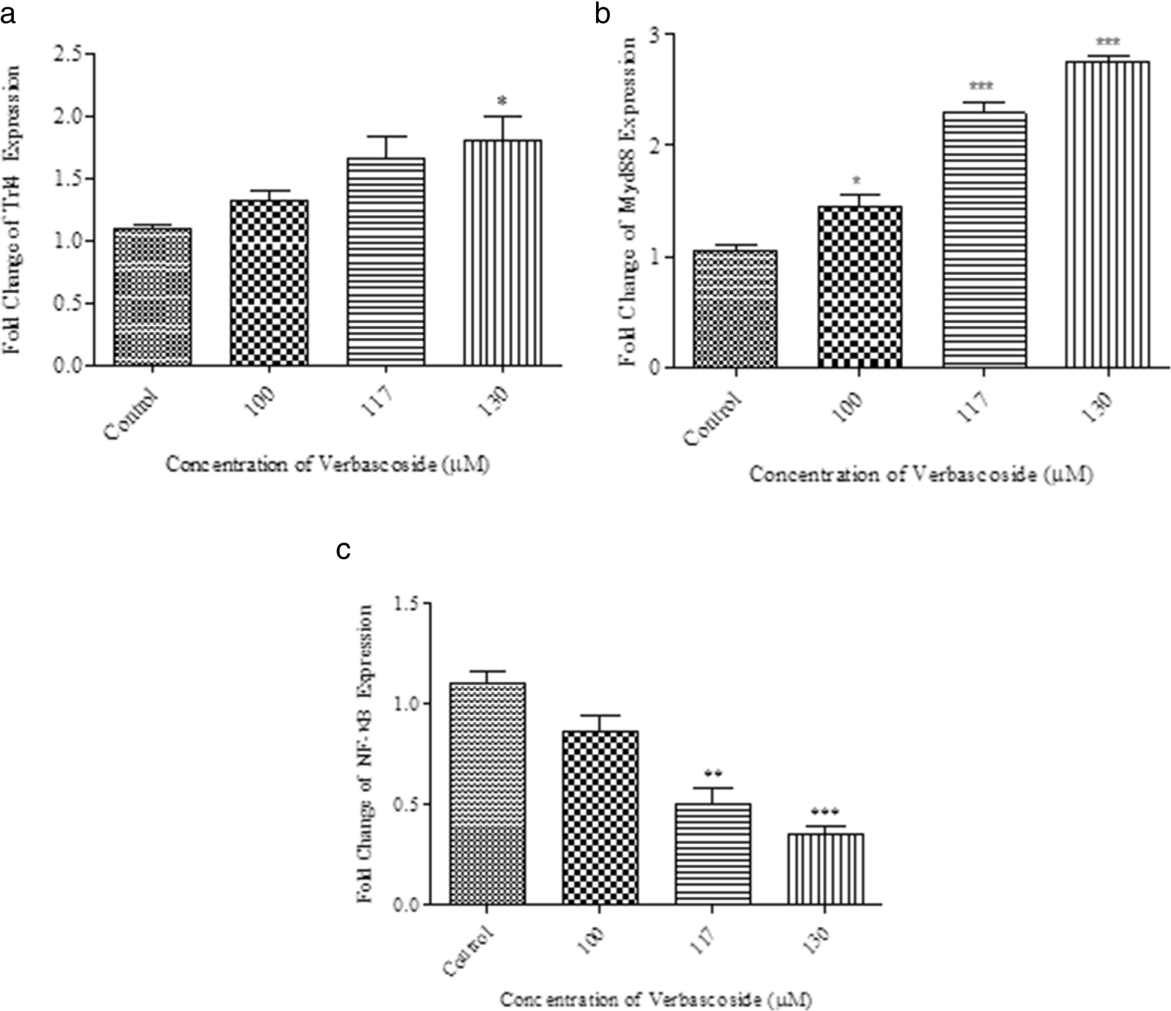
Gene expression alteration of Nf-κB, *MyD88* and, TLR4 by verbascoside in 4 T1 cells. **a** Shows verbascoside treatment led to overexpression of TLR4 but was only significant at 130 μM dose (*P* < 0.05); 1.78 ± 0.32 fold. **b** Demonstrates verbascoside effectively increased the expression of *MyD88* in a dose-dependent manner; 1.44 ± 0.20 fold at 100 μM (*P* < 0.05), 2.29 ± 0.17 at 117 μM (*P* < 0.001) and, 2.79 ± 0.07 at 130 μM dose (*P* < 0.001). **c** Exhibits the effect of verbascoside treatment on the expression of NF-κB in 4 T1 cells, verbascoside significantly reduced the expression of NF-κB by 0.50 ± 0.13 fold at 117 μM (*P* < 0.01) and 0.35 ± 0.05 fold at 130 μM concentration (*P* < 0.001). (*N* = 3) *** *P* < 0.001, ** *P* < 0.01, * *P* < 0.05 compared to control

To determine verbascoside by which pathway initiate apoptosis in *4 T1* cells, we also investigated the expression of *TLR4*, *MyD88*, and *NF-κB* under treatment at the mRNA level. Verbascoside slightly increased the expression of *TLR4* at mRNA level but it was not significant at 100 μM and 117 μM doses, however, the change was significant at the dose of 130 μM (*P* < 0.05) (Fig. 3-a).

Also, verbascoside effectively increased the expression of *MyD88* gene in a dose-dependent manner at all three doses. The expression change was slightly significant at 100 μM dose (*P* < 0.05) but was more significant at 117 and 130 μM doses (for both *P* < 0.001) (Fig. 3-b).

*NF-κB* gene expression was down-regulated by verbascoside dose dependently which was significant at 117 μM (*P* < 0.01) and 130 μM concentration (*P* < 0.001) (Fig. 3-c).

### Flow cytometry demonstrated apoptosis induction by verbascoside

**Fig. 4:**
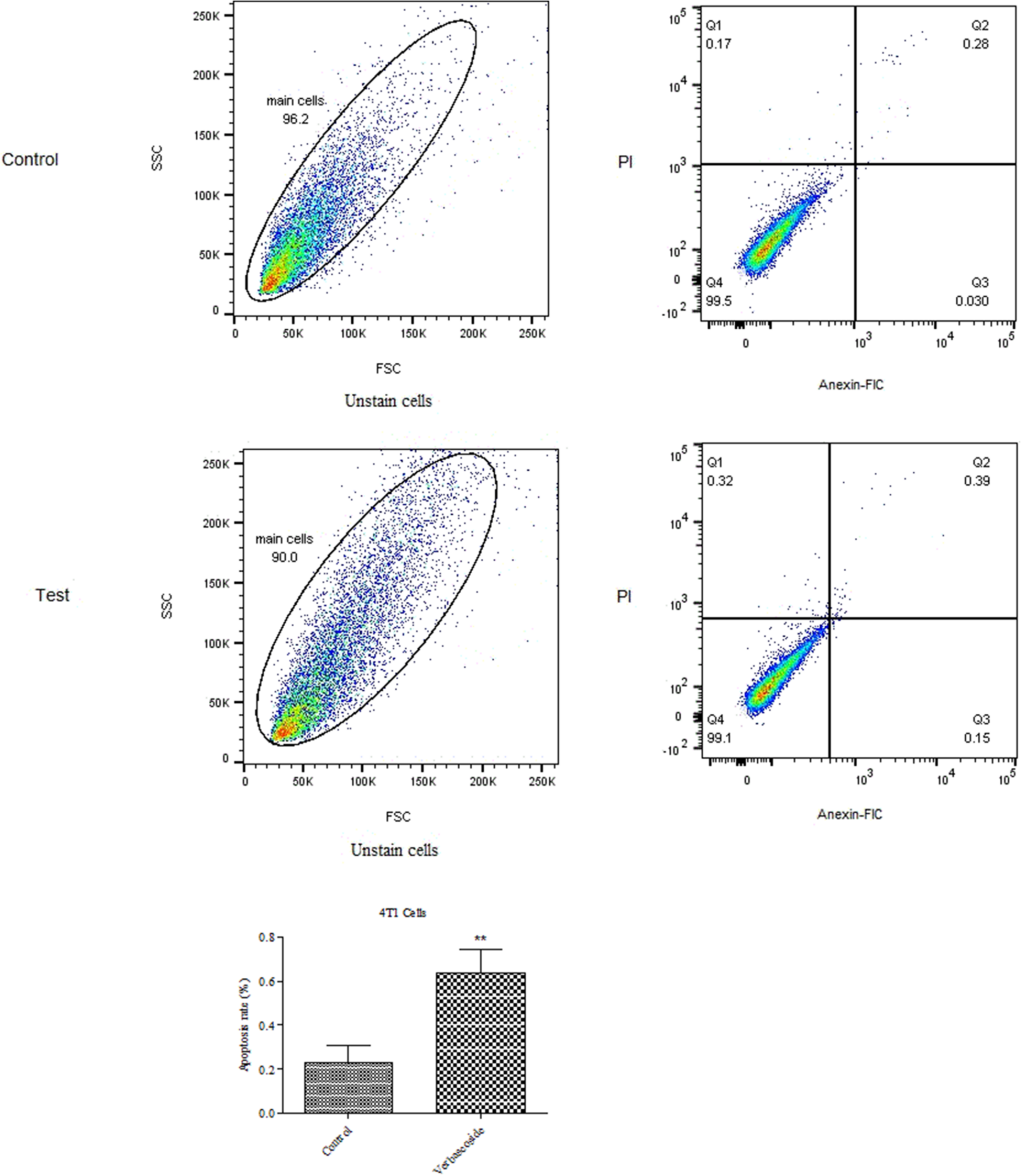
Flow cytometry examination showed that verbascoside induces apoptosis in 4 T1 cells (*P* < 0.01 N = 3). Control boxes refer to cells with no verbascoside treatment. Test boxes are showing cells under verbascoside treatment. Column graphs are showing the percentage of apoptotic cells. ** *P* < 0.01, * *P* < 0.05 compared to control

To ascertain the apoptotic effect of verbascoside on *4 T1* cells, we also performed *PI* and *Annexin-V* double-staining followed by flow cytometry. *4 T1* cells were treated with verbascoside (117 μM) for 24 h and then stained and flow cytometry was conducted. Results showed that verbascoside slightly increased the number of apoptotic cells compared to untreated. However, the percentage of necrosed cells were slightly higher in treated cells compared to untreated cells. Figure 4 shows the rate of apoptotic cells in verbascosid group comparison to the control group. Verbascoside increased the number of apoptotic cells by 0.64 *±* 0.6% compared to the control group (0.22 *±* .0.04) which shows was significant (*P* < 0.01).

## Discussion

Since now, various biological functions of polyphenol compounds have been reported such as anti-oxidant, anti-inflammatory [23], and more importantly anti-tumor [24] activity. Verbascoside also belongs to this class of compounds and is an active phytochemical of *Cistanches Herba* of Orobanchaceae family. Some reports have shown this compound has anti-tumor and apoptosis-inducing features on glioblastoma [25], colorectal [26] and, head and neck carcinomas [27].

Our findings showed verbascoside has anti-tumor activity against breast cancer cells by initiating apoptosis cascade followed by activating *MyD88* pathway and reducing *NF-κB* at mRNA level with no toxic effect on normal cells. Our results from gene expression analysis and also flow cytometry revealed that verbascoside initiates apoptosis in breast cancer cells.. Also, we found that verbascoside at 130 μM concentration is able to enhance the expression of TLR4.

Anti-cancer feature of verbascoside against various cancers has been reported [25, 27]. As *MTT* assay results showed that verbascoside is toxic to *4 T1* cells, we investigated the underlying mechanism. In this study, we measured the expression of genes related to apoptosis cascade in *4 T1* cells. We showed verbascoside dose-dependently increased the *Bax/Bcl-2* ratio as a sign of apoptosis. Also, we showed overexpression of *caspase-3* upon verbascoside treatment in *4 T1* cells. *Bax* and *Bcl-2* are major determiners of cell survival or apoptosis. *Bcl-2* is a member of the *Bcl2* family of genes which confers survival to cancer cells [28, 29] whereas *Bax* is responsible for apoptosis initiation and eventually cell death [29]. *Bax* inhibits the activity of *Bcl-2* upon binding and forming a heterodimer with, consequent of apoptosis stimuli [29]. In this condition, *Bax* by binding to mitochondrial membrane disrupts its integrity which in turn results in cytochrome C release into the cytoplasm. Cytochrome C by forming apoptosome with *procaspase-9* and apoptotic protease activating factor 1, cleaves *caspase-3* and initiates apoptosis [30].

Our results of *PI* and *annexin-V* staining also confirmed apoptosis initiation by verbascoside. DNA fragmentation subsequent of apoptosis is detectable by *PI* staining due to to the ability of *PI* in binding to DNA molecules. In addition, *annexin-V* binds to phosphatidylserine (*PS*) on the surface of cells [31]. Increase in the content of PS on the cell surface is a well-documented indicator of apoptosis in cells. Taken together, increased fluorescence in cells stained with *PI* and *annexin V* reflects apoptosis condition [32].

Myeloid differentiation primary response 88 (*MyD88*) pathway is able to mediate apoptosis which is a downstream molecule of *TLR4* [33]. Inhibition of *NF-κB* after *MyD88* activation induces apoptosis in cancer cells [34]. Our findings showed *MyD88* and *TLR4* were overexpressed by verbascoside. It seems that verbascoside by increasing the *TLR4* at the cell surface acts on *MyD88*, as its downstreatm molecule, [35] and induces apoptosis by this mechanism. In addition, we observed *NF-κB* is downregulated which may be due to the activation of *MyD88* [34], or may be the direct effect of verbascoside [36]. Verbascoside has been reported to prevent *IκBα* degradation which keeps *NF-κB* in its inactive state in the cytoplasm of cells [27, 37]. Therefore, *MyD88* upon activation by upper regulatory signals initiates apoptosis pathway through FAS-associated death domain protein (*FADD*) which consequently affects procaspase-8 and apoptosis cascade [38]. Verbascoside can activate *HIPK2* [9] which in turn directly inhibits *NF-κB* to promote apoptosis [10].

In conclusion, herein, we showed that while verbascoside is safe and nontoxic for normal cells could be a a natural anti-tumor compound against breast cancer cells.

## Data Availability

The data that support the findings of this study are available on request from the corresponding author. The data are not publicly available due to privacy or ethical restrictions.
